# Low-dose urokinase thrombolytic therapy for patients with acute intermediate-high-risk pulmonary embolism: A retrospective cohort study

**DOI:** 10.1371/journal.pone.0248603

**Published:** 2021-03-26

**Authors:** Cuilian Weng, Xincai Wang, Long Huang, Xingsheng Lin, Qinghua Liu

**Affiliations:** 1 Department of Intensive Care Unit, Fujian Provincial Hospital South Branch, The Shengli Clinical Medical College of Fujian Medical University, Fuzhou, Fujian Province, China; 2 Department of Otorhinolaryngology, Fujian Provincial Hospital, The Shengli Clinical Medical College of Fujian Medical University, Fuzhou, Fujian Province, China; Klinikum Guetersloh, GERMANY

## Abstract

**Introduction:**

Patients at intermediate-high risk of developing a pulmonary embolism (PE) are very likely to experience adverse outcomes, such as cardiovascular instability and death. The role of thrombolytic therapy in intermediate-high-risk PE remains controversial.

**Objectives:**

This study aimed to determine the efficacy and safety of low-dose urokinase (UK) thrombolytic therapy for intermediate-high-risk PE.

**Patients and methods:**

This retrospective study included 81 consecutive patients with intermediate-high-risk PE from two centers. Patients received low-dose UK or low-molecular-weight heparin (anticoagulant therapy group). The efficacy outcomes were mortality, computed tomography pulmonary angiography (CTPA)-confirmed absorption, and dyspnea. Safety was assessed as the incidence of bleedings.

**Results:**

The in-hospital mortality, 9-month mortality, and long-term mortality at the last follow-up were comparable for the low-dose UK group and the anticoagulant therapy group (6.45% vs. 0%, *p* = 0.144, 9.68% vs. 8.16%, *p* = 0.815, and 12.90% vs. 12.24%, *p* = 0.931, respectively). CTPA-confirmed absorption at one month after admission was higher in the low-dose UK group than in the anticoagulant therapy group (*p* = 0.016). The incidences of short-term dyspnea at discharge and long-term dyspnea at the last follow-up were lower in the low-dose UK group than in the anticoagulant therapy group (27.59% vs. 52%, *p* = 0.035, 33.33% vs. 58.14%, *p* = 0.043, respectively). No major bleeding occurred. The incidence of minor bleeding was not significantly different between the two groups (3.23% vs. 6%, *p* = 0.974).

**Conclusion:**

In intermediate-high-risk PE, a low-dose UK might increase CTPA-confirmed absorption and improve short-term and long-term dyspnea without affecting mortality or increasing the bleeding risk.

## Introduction

Pulmonary embolism (PE) is the third most common cardiovascular disease, after myocardial infarction and stroke. PE can cause serious disability or even death. In epidemiological studies, the annual incidence of PE ranges from 39 to 115 per 100,000 individuals [1]. Additionally, about 7–11% of all patients with acute PE die within 3 months of the diagnosis [1–3].

The 2019 European Society of Cardiology (ESC) guidelines have classified PE into 3 groups: high-risk PE, intermediate-risk PE (including intermediate-high-risk PE and intermediate-low-risk PE), and low-risk PE [1]. High-risk PE is characterized by overt hemodynamic instability, and systemic thrombolytic therapy is strongly recommended [1, 4]. Intermediate-high-risk PE is defined as the presence of both right ventricular (RV) dysfunction and elevated cardiac laboratory biomarkers, but no systemic hypotension. Intermediate-low-risk PE is defined as either echocardiographic-confirmed RV dysfunction or elevated troponin levels(or normal RV function and troponin levels with a simplified Pulmonary Embolism Severity Index≥1) without hemodynamic instability.

Due to the RV dysfunction and myocardial injury induced by acute RV pressure overloads, patients with intermediate-high-risk PE are at a relatively higher risk of developing adverse outcomes, such as death and other serious events (cardiovascular instability, hemodynamic collapse, need of intubation, etc.), compared to patients with intermediate-low-risk PE [5]. In principle, thrombolytic therapy should promptly reduce the RV pressure and improve RV function in intermediate-high-risk PE. However, the role of thrombolytic therapy in treating patients with intermediate-high-risk PE remains controversial [6].

Few studies have focused on the population of patients with intermediate-high-risk PE [7–9]. Meyer et al showed that, among patients with intermediate-risk PE, tenecteplase therapy caused less hemodynamic decompensation than placebo treatment [odds ratio (OR): 0.30; 95% confidence interval (CI): 0.14 to 0.68; *p* = 0.002] [7]. However, tenecteplase thrombolytic therapy increased the risk of major extracranial bleeding and stroke (*p*<0.001 and *p*<0.003, respectively) [7]. Nevertheless, a meta-analysis of seven studies on acute intermediate-risk PE (n = 1631 patients) indicated that the incidences of major bleeding were not significantly different between the thrombolytic group and the anticoagulant group (OR: 2.07; 95% CI: 0.60–7.16; *p* = 0.25) [10]. Although the 2019 guidelines indicate that anticoagulation therapy is the mainstay of treatment, early management with a reperfusion strategy remains an issue of debate in intermediate-high-risk PE [11].

Some low-dose thrombolytic therapies have been used for local treatments, such as catheter-directed thrombolysis, and none have demonstrated an increased risk of bleeding [12, 13]. We reasoned that systemic low-dose thrombolysis might be safer than full-dose thrombolysis, because a low dose would provide lower risk of incurring major or fatal bleeding. Therefore, we hypothesized that systemic low-dose thrombolytic therapy might be a candidate for the early treatment of intermediate-high-risk PE. To date, data are limited on the use of low-dose thrombolysis in intermediate-high-risk PE. Few studies have focused on the efficacy, safety, and long-term effects of urokinase (UK) thrombolysis in patients with PE. Therefore, the current study aimed to investigate the clinical efficacy and safety of low-dose UK thrombolysis in patients with intermediate-high-risk PE.

## Methods

### Study design

This retrospective cohort study was performed in two tertiary hospitals (Fujian Provincial Hospital, from March 1, 2013, to June 30, 2019, and Fujian Provincial Hospital South Branch, from May 1, 2015, to June 30, 2019). Data on all enrolled patients were extracted from medical records and evaluated by two independent clinical physicians (WC and XW). A third researcher (XL) adjudicated any difference in interpretation between the two primary reviewers. The anonymity of the medical data was strictly monitored by one physician (XL), who had access to information on the individual participants during and after data collection. To protect patient privacy, the data were anonymized with a code that did not include any information on patient identity. This study was approved by the Ethics Committee of Fujian Provincial Hospital. Patient informed consent was waived, due to the retrospective, anonymized nature of the study.

### Identification of the study population

All consecutive adult patients (≥18 years old) that were diagnosed with PE were screened for the study. Patients eligible for this study had to meet all the following criteria [9]: (1) onset of symptoms within the prior 14 days; and (2) an intermediate-high-risk PE diagnosis, according to the 2019 ESC guidelines. To confirm RV dysfunction, patients had to meet at least one of the following echocardiographic criteria: a RV end-diastolic diameter (RVEDD) >30 mm; a right/left ventricular end-diastolic diameter ratio (RVEDD/LVEDD) >0.9; hypokinesis of the RV free wall; or tricuspid systolic velocity >2.6 m/s. When an echocardiograph was not available, RV dysfunction was assessed with CT by measuring the minor axis of the right and left ventricles in the transverse plane and calculating the RV diameter/LV diameter ratio (ratios >0.9 = RV dysfunction). Myocardial injury was confirmed, when the cardiac troponin I level exceeded the upper limit of normal. Exclusion criteria were: (1) patients under 18 years old; (2) a diagnosis of high-risk PE, intermediate-low-risk PE, or low-risk PE; (3) intermediate-high-risk PE treated with other therapies, such as reteplase-mediated thrombolysis or catheter-directed thrombolysis.

### Treatment

All patients were treated with either systemic low-dose UK combined with low-molecular-weight heparin (LMWH; low-dose UK group) or LMWH alone (anticoagulant therapy group). The low-dose UK group received an intravenous administration of 10,000 U/kg UK in a 2-h infusion, once per day for 5 days [8, 14]. These patients received LMWH after the UK thrombolytic therapy. In the anticoagulant therapy group, all patients received only LMWH. Then, warfarin or other oral anticoagulants were initiated in two groups. Warfarin therapy and other oral anticoagulants were administered according to ESC guidelines.

### Data collection, outcome assessment, and follow-up

The epidemiological, demographic, clinical, laboratory, radiologic, and outcome data were extracted from electronic medical records and from telephone contacts. Patient follow-ups were performed by evaluating medical records and by contacting patients by telephone.

The primary efficacy outcomes were: mortality, CTPA-confirmed absorption, short-term dyspnea (defined as dyspnea at discharge) according to medical records, and long-term dyspnea (defined as dyspnea at the last follow-up, over a period of at least more than 9 months) according to the New York Heart Association (NYHA) Functional Class. All patients underwent CTPA examinations by a single experienced radiologist before receiving treatment. CTPA examinations were repeated at one month after admission. CTPA-confirmed absorption was graded as follows: grade 1: a pulmonary defect area reduced by <25%; grade 2: a pulmonary defect area reduced by >25%, but <50%; grade 3: a pulmonary defect area reduced by >50%, but <75%; grade 4: a pulmonary defect area reduced by >75%.

The secondary efficacy outcomes were: the need for endotracheal intubation; the need for therapy escalation (defined as either a catecholamine infusion, due to shock, or cardiopulmonary resuscitation); the incidences of recurrent and chronic PE; and daily self-care ability, evaluated with the modified Barthel Index at the last follow-up. Recurrent and chronic PE were confirmed with either ventilation–perfusion lung scans or CTPA.

The primary safety outcome was major bleeding, and the secondary safety outcome was minor bleeding. Major bleeding was defined as fatal bleeding, hemorrhagic stroke, or a drop in the hemoglobin concentration by at least 4 g/dL. All patients were followed for >11 months. The last follow-up was on May 31, 2020. Only one patient in the anticoagulant therapy group was excluded from the follow-up after one month, due to the need for a CTPA re-examination.

### Statistical analysis

Statistical analyses were performed with SPSS 20 (SPSS Inc, Chicago, IL). Continuous variables are expressed as the mean and standard deviation (SD) or the median and interquartile range (IQR). Continuous variables were evaluated with the T test or the Wilcoxon test, as appropriate. Categorical variables are expressed as the counts and the percentage in each category. Categorical variables were evaluated with the Chi-square test, Fisher’s exact test, or Mann-Whitney U test, as appropriate. The Mann-Whitney U test was used to evaluate the rand data from the CTPA. The time to death was analyzed with Kaplan-Meier plots, and comparisons between groups were evaluated with log-rank tests. To explore the risk factors associated with CTPA-confirmed absorption, we used an ordinal logistic regression model. The hazard ratio (HR) and 95% CI for long-term dyspnea at the last follow-up were calculated with a Cox proportional hazards model. Statistically significant variables were included into the final models. A two-sided α <0.05 was considered statistically significant.

## Results

### Demographic and clinical characteristics

**Fig 1:**
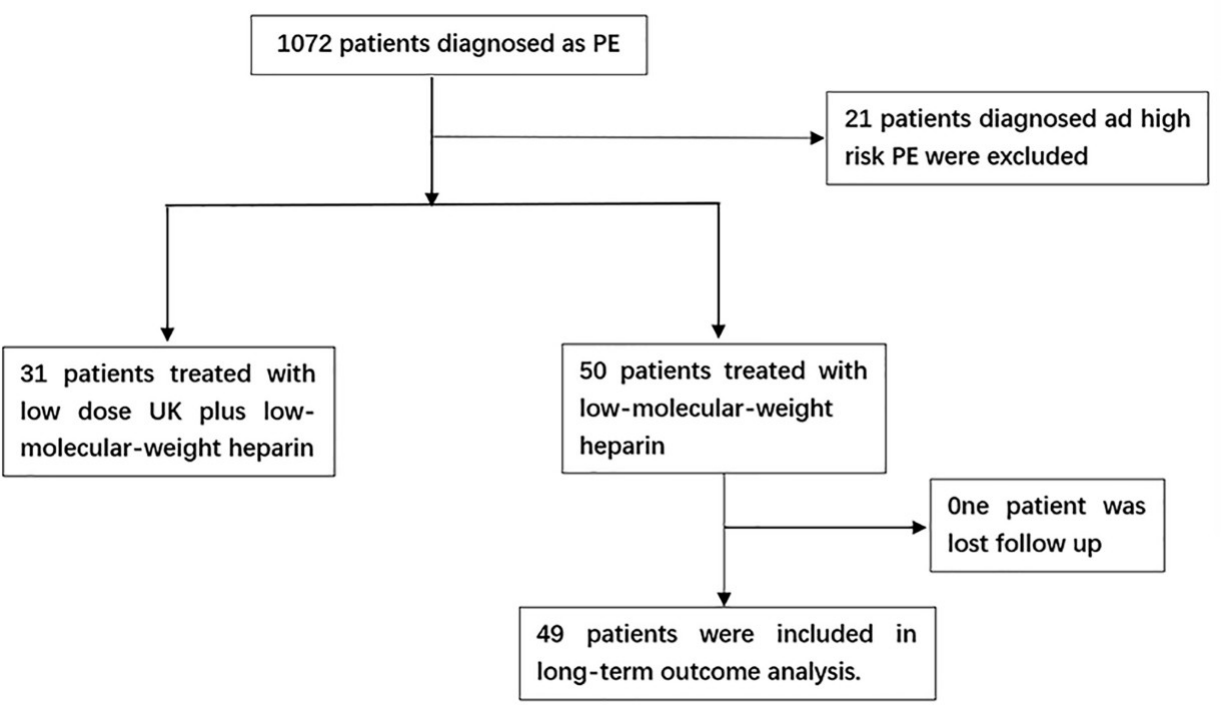
Patient selection process.

About 1072 patients diagnosed with PE were screened in this cohort study. Among these, 81 had confirmed acute intermediate-high-risk PE (Fig 1). Of these, 31 patients were treated with low-dose UK and 50 patients were treated with anticoagulant therapy. Only one patient (in the anticoagulant therapy group) was lost to follow-up one month later, due to a CTPA re-examination. The mean (SD) follow-up times were 45.20 (25.89) months in the low-dose UK group and 38.96 (21.49) months in the anticoagulant therapy group (*p* = 0.244).

**Table 1** shows the demographics, medical history, and clinical status of all patients at baseline. We found no significant differences in baseline characteristics between groups.

**Table 1:** Demographic characteristics of patients at baseline.

Characteristic	Low-dose UK group, N = 31		Anticoagulant therapy group, N = 50		P
	N	%	N	%	
Age, years; mean (SD)	62.9 (10.7)		67.2 (12.4)		0.118
Males	16	51.61	19	38.00	0.229
Smoking	6	19.35	11	22.00	0.776
Drinking	2	6.45	3	6.00	0.935
**Comorbidity**					
Chronic lung diseases	1	3.23	4	12.90	0.694
Hypertension and CAD	13	41.94	18	36.00	0.593
Nervous system diseases	0	0	1	2.00	0.428
Diabetes	4	12.90	3	6.00	0.504
Cancer	1	3.23	4	8.00	0.694
Major operation within 4 weeks*	4	12.90	1	2.00	0.132
Immobilization within 4 weeks	7	22.58	8	16.00	0.459
Use of hypercoagulants, glucocorticoids, contraceptives, or estrogens	1	3.23	1	2.00	1.000
Unilateral lower extremity edema or pain within 4 weeks	3	9.68	9	18.00	0.482
Great saphenous vein varicosis	3	9.68	7	14.00	0.820

Values are the number of patients (%), unless otherwise indicated. CAD, Chronic coronary artery disease.

* All patients who underwent major operation were received low dose UK thrombolysis 3 weeks after surgery.

### Baseline laboratory, radiographic, and ultrasound findings

Among the signs and symptoms, we found that systolic blood pressure (SBP) was lower in the low-dose UK group than in the anticoagulant therapy group (mean [SD] SBP: 118.71 [18.30] vs. 127.66 [19.18], *p* = 0.041). The laboratory findings were not significantly different between the two groups (Table 2). The PE diagnosis was confirmed with CTPA in all patients. Bilateral PE was significantly more common in the low-dose UK group than in the anticoagulant therapy group (96.77% vs. 80% *p* = 0.044). The two groups were not significantly different in any other radiographic or ultrasound findings (Table 2).

**Table 2:** Baseline clinical, laboratory, and radiographic findings.

Characteristic	Low-dose UK group, N = 31		Anticoagulant therapy group, N = 50		P
	N	%	N	%	
**Symptom**					
Cough	10	32.26	18	36.00	0.731
Hemoptysis	1	3.23	3	6.38	0.974
Dyspnea	30	96.77	44	88.00	0.337
Chest tightness	22	70.97	28	56.00	0.178
Chest pain	10	47.62	13	35.14	0.544
Edema in lower extremity	5	16.13	12	24.00	0.398
Lower extremity pain	4	12.90	3	6.00	0.504
**Signs**					
HR beats/min, mean (SD)	22.68 (3.360)		21.56 (3.51)		0.161
SBP (mmHg), mean (SD)	118.71 (18.30)		127.66 (19.18)		0.041
PaO_2_/FiO_2_					
PaO_2_/FiO_2_≥200	17	54.84	27	54.00%	0.941
**Laboratory findings**					
Hb g/L, mean (SD)	133.00 (23.44)		131.86 (21.57)		0.824
PLT 10^9^/L, mean (SD)	194.68 (66.56)		189.98 (53.462)		0.728
Creatinine μmol/L, mean (SD)	74.42 (23.24)		75.78 (19.25)		0.776
BNP ng/L, mean (SD)	3232.32 (2581.21)		4228.62 (4358.70)		0.253
cTnI ng/mL, mean (SD)	0.72 (1.81)		0.45 (0.948)		0.384
d-dimer μg/L, mean (SD)	11.24 (7.72)		11.00 (8.79)		0.897
**Radiographic findings**					
Bilateral pulmonary embolism	30	96.77	40	80.00	0.044
Pulmonary embolism involving the main pulmonary artery	23	74.19	36	72.00	0.829
Pulmonary embolism involving three lung lobes	29	93.55	41	82.00	0.254
Vein thrombosis in both lower extremities	2	6.45	9	18.00	0.254
Deep vein thrombosis only in calves	5	16.13	17	34.00	0.079
Deep vein thrombosis in entire femoral vein	6	20.00	9	18.00	0.879

Values are the number of patients (%), unless otherwise indicated. HR, heart rate; SBP, Systolic blood pressure; PaO_2_, partial pressure of oxygen; FiO_2_, fraction of inspired oxygen; Hb, Hemoglobin; PLT, blood platelets; BNP, B-type natriuretic peptide; cTnI, cardiac troponin I.

### Clinical outcomes

#### Mortality and the need for therapy escalation

**Fig 2:**
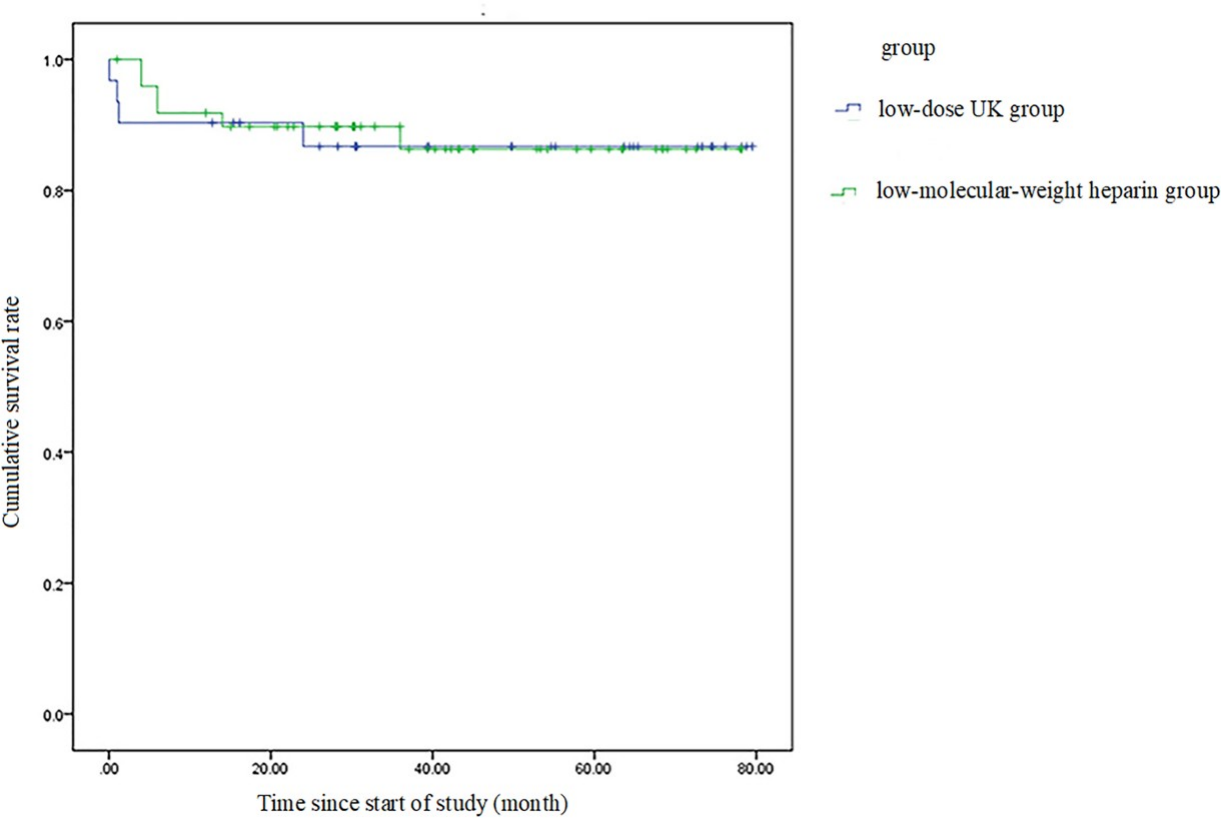
The Kaplan–Meier estimates of cumulative survival rate. Patients with intermediate-high-risk pulmonary embolism were treated with either low-dose urokinase (UK) plus low-molecular-weight heparin (LMWH) or LMWH alone; p=0.894, based on the log-rank test.

The two groups showed no significant differences in the in-hospital mortality, 90-day mortality, six-month mortality, nine-month mortality, or mortality at the last-follow-up (Table 3). A Kaplan–Meier analysis showed that the probability of event-free survival at the last follow-up was not significantly different between groups (*p* = 0.894 log-rank test; Fig 2). An escalation in therapy and endotracheal intubation were required in two patients in the low-dose UK group (6.45%) and no patient in the anticoagulant therapy group (*p* = 0.144). No patient required secondary rescue thrombolysis (Table 3).

**Table 3:** Efficacy and safety outcomes.

Characteristic	Low dose UK group, N = 31		Anticoagulant therapy group, N = 50		P
**Outcome**	N	%	N	%	
Need of mechanical ventilation	2	6.45	0	0	0.144
Need of therapy escalation	2	6.45	0	0	0.144
Need of high-flow oxygen	24	77.42	36	72.00	0.589
Organ damage in hospital, mean (SD)	2.01 (0.473)		1.98 (0.319)		0.188
Hospital length of stay, days, median [IQR]	13 [8–19]		14 [10–17]		0.853
**CTPA-confirmed absorption**	N = 30		N = 50		
0–25% absorption	0	0	2	4.00	
25–50% absorption	4	13.33	13	26.00	
50–75% absorption	10	33.33	21	42.00	
>75% absorption	16	53.33	14	28.00	
					0.016*
**Follow-up, months,** mean (SD)	45.20 (25.89)		38.96 (21.49)		0.244
**All-cause death**					
	N = 31		N = 50		
In-hospital mortality	2	6.45	0	0.00%	0.144
	N = 31		N = 49		
90-Day mortality	3	9.68	0	0	0.106
6-month mortality	3	9.68	4	8.16	0.815
9-month mortality	3	9.68	4	8.16	0.815
Last-follow-up mortality	4	12.90	6	12.24	0.931
**Causes of death**	N = 31		N = 49		
PE	2		0		
Sudden death	1		1		
Heart failure	0		3		
Cancer	1		1		
Stroke	0		1		
**Dyspnea**					
Dyspnea at discharge	N = 29		N = 50		
	8	27.59	26	52.00%	0.035
Dyspnea at last follow-up	N = 27		N = 43		
	9	33.33	25	58.14	0.043
NYHA Class I	18	55.56	18	81.40	
NYHA Class II	7	37.04	19	13.95	
NYHA Class III	2	7.41	5	4.65	
NYHA Class IV	0	0	1	2.33	
					0.049*
**Self-care ability at last follow up**	N = 27		N = 43		
	27	100	42	97.67	1.000
	N = 27		N = 43		
**Recurrent PE**	0	0	2	4.65	0.519
**CTEPH**	0	0	1	2.33	1.000
**Bleeding**	N = 31		N = 50		
Intracranial hemorrhage	0	0	0	0	
Gastrointestinal bleeding	0	0.00%	0	0.00%	
Massive hemoptysis	0	0.00%	0	0.00%	
Urinary tract bleeding	1	3.23%	1	2.00%	1.000
Oral and nasal bleeding	0	0.00%	2	4.00%	0.522

Values are the number of patients (%), unless otherwise indicated. CTPA, computed tomography pulmonary angiography; NYHA, New York Heart Association Functional Class for evaluating physical performance; PE, pulmonary embolism; CTEPH, Chronic thromboembolic pulmonary hypertension; *Mann-Whitney U test was used to evaluate rand data.

#### CTPA-confirmed absorption

The effect of thrombolysis or anticoagulant therapy was evaluated with CTPA at one month after admission. CTPA confirmed that the degree of thrombus absorption was significantly higher in the low-dose UK group than in the anticoagulant therapy group (p = 0.016, Table 3). An ordinal logistic regression model analysis showed that the grade of CTPA-confirmed absorption was significantly associated with the administration of low-dose UK treatment (OR: 2.76, 95% CI: 1.09–7.03), the presence of bilateral PE (OR: 7.37, 95% CI: 1.88–28.94), and the presence of PE without main pulmonary artery involvement at admission (OR: 5.50, 95% CI: 1.83–16.55).

#### Dyspnea

Compared to the anticoagulant therapy group, the low-dose UK group reported lower incidences of both short-term dyspnea at discharge (27.59% vs. 52%, *p* = 0.035) and long-term dyspnea at the last follow-up (33.33% vs. 58.14%, *p* = 0.043; Table 3). A Cox proportional hazards model indicated that the odds of developing long-term dyspnea were higher among patients treated with anticoagulant therapy (HR: 3.508, 95% CI: 1.531–8.039) and those with hypertension comorbidity in coronary artery disease (HR: 2.715, 95% CI: 1.324–5.563).

Recurrent PE occurred in two patients in the anticoagulant therapy group (*p* = 0.519). Only one patient in the anticoagulant therapy group was diagnosed with chronic thromboembolic pulmonary hypertension (CTEPH; *p* = 1.000; Table 3). That patient did not undergo surgical treatment and survived to the last follow-up. The ability to perform self-care activities was not significantly different between the two groups (*p* = 1.000; Table 3).

#### Safety outcome

No major hemorrhage or major cranial bleeding occurred. The incidence of minor bleeding was similar between the two groups (3.23% vs. 6%, *p* = 0.974; Table 3).

## Discussion

This retrospective cohort study showed that, among patients with intermediate-high-risk PE, low-dose UK treatment and anticoagulant therapy were associated with similar outcomes regarding in-hospital mortality, 90-day all-cause mortality, 6-month all-cause mortality, 9-month all-cause mortality, and last follow-up all-cause mortality. However, compared to anticoagulant therapy, low-dose UK treatment was associated with an increase in the absorption of intravascular thrombosis. Low-dose UK treatment also improved short-term and long-term dyspnea, compared to anticoagulation therapy. Two cases of recurrent PE and one case of chronic thromboembolic pulmonary hypertension occurred in the anticoagulant therapy group and none occurred in the low-dose UK group, though the difference was not significant between the two groups. Low-dose UK treatment did not increase the risk of major or minor bleeding events, compared to anticoagulation therapy.

The incidence of intermediate-high-risk PE was 7.5% in our cohort study. This prevalence was lower than that of a previous study, which showed a prevalence as high as 30% [15], but it was similar to the prevalence reported by Mirambeaux et al. (n = 97/1015, 9.6%; 95%CI: 7.8–11.5%) among normotensive patients with acute PE [16]. This discrepancy might be explained, in part, by the study design. In the present study and the Mirambeaux et al. study, we identified patients with more severe intermediate-risk acute PE with the combination of a positive simplified Pulmonary Embolism Severity Index and echocardiographic evidence of RV dysfunction and myocardial injury, as recommended by the 2014 and 2019 ESC guidelines [16]. Clinical practice guidelines suggest that patients with intermediate-high-risk PE should be closely monitored, and prompt treatment should be given when hemodynamic decompensation occurs [1, 4]. Indeed, studies have shown that patients diagnosed with intermediate-high-risk PE had a greater risk of short-term death compared to patients with intermediate-low-risk PE [15, 16]. Becattini et al. [15] showed that, in a cohort of 906 patients, death due to PE within 30 days occurred in 0.5% of patients at “low-risk” (95% CI: 0–1.5), 2.1% of patients at “intermediate-low-risk” (95% CI: 0.6–3.6), 4.8% of patients at “intermediate-high-risk” (95% CI: 2.2–7.3), and 15.2% of patients at “high-risk” (95% CI: 8.4–22.1). Mirambeaux et al. [16] showed that 23 of 97 patients with intermediate-high-risk PE (24%; 95% CI: 16–33%) experienced adverse outcomes, such as PE-related mortality, hemodynamic collapse, or recurrent PE [16]. Therefore, among patients with intermediate-high-risk PE, prompt reduction of pulmonary artery resistance might reduce the RV pressure, improve RV function, and reduce myocardial injury. Accordingly, thrombolytic therapy might be the therapy of choice for intermediate-high-risk PE, because intravascular thrombosis can be resolved rapidly.

In our cohort study, the in-hospital morality and the 30-day all-cause mortality attributed to PE were both 2.50% in all patients. These results indicated that low-dose UK treatment did not significantly reduce short-term mortality (i.e., in-hospital morality and 30-day all-cause mortality), compared to anticoagulant therapy, in patients with intermediate-high-risk PE. These findings were similar to those from a previous study, which showed that, compared to placebo, tenecteplase did not improve 30-day mortality (3.2% vs. 2.4%, *p* = 0.42) [7]. In the present cohort study, two patients in the low-dose UK group died during hospitalization, compared to none in the anticoagulant therapy group. This finding could be attributed to the fact that more patients in the low-dose UK group had PE involvement in both lungs (*p* = 0.044) and a lower average systolic blood pressure on admission (*p* = 0.041), compared to the anticoagulation group. The mean follow-up, calculated over all patients in this cohort study, was 41.3 months. The 9-month all-cause mortality and last follow-up all-cause mortality were 8.75% and 12.5%, respectively. Thus, eight patients died over the long term, including three patients that died of heart failure, two patients that died of cancer, two patients that experienced a sudden unexplained death, and one patient that died of ischemic stroke. These results were similar to those reported in the study by Konstantinides et al., where after 30 days, most deaths were caused by an underlying disease or comorbidity [17].

However, our results indicated that low-dose UK fibrinolytic treatment accelerated CTPA-confirmed absorption at one month after admission. The ordinal logistic regression model showed that low-dose UK treatment, bilateral PE, and PE without main pulmonary artery involvement at admission were associated with higher odds of CTPA-confirmed absorption.

In the present study, the rates of dyspnea at admission were comparable between the two groups (96.7% vs. 88%, *p* = 0.337). At discharge, dyspnea was observed in a lower proportion of patients in the low-dose UK group than in the anticoagulant therapy group (27.59% vs. 52%, *p* = 0.035). Moreover, previous studies have reported that, in the long term, persistent symptoms, reduced exercise capacity, and impaired quality of life were frequently observed in survivors of acute PE [18–21]. In a post hoc analysis of 219 survivors of acute intermediate-risk PE in the Pulmonary Embolism Thrombolysis trial, post-PE impairments were observed in 23 patients [20]. In another prospective cohort study of 127 patients diagnosed with ‘submassive’ PE, at 6 months, 17% had RV dysfunction, 17% had functional limitations, and 8% had both [22]. In our study, the long-term data from the last follow-up showed that dyspnea occurred less frequently in the low-dose UK group (33.33%) compared to the anticoagulant therapy group (58.14%, *p* = 0.043). Our study also suggested that, compared to the low-dose UK group, the anticoagulant therapy group had a 3.508-fold higher risk of long-term dyspnea (HR: 3.508, 95% CI: 1.531–8.039). Additionally, compared to patients without hypertension or CAD, patients with hypertension or CAD had a 2.854-fold higher the risk of long-term dyspnea (HR: 2.715, 95% CI: 1.324–5.563), based on a Cox proportional hazards model.

Our cohort study showed bleeding results similar to those found in a meta-analysis by Xu et al. [10] and other studies [22–24]. We found that low-dose UK treatment did not increase the risk of major or minor bleeding events, compared to anticoagulant therapy.

In the anticoagulant therapy group, recurrent PE occurred in two patients, and CTEPH occurred in one patient. In contrast, no patients experienced recurrent PE or CTEPH in the low-dose UK group. Nevertheless, these results were not significantly different. In this cohort study, we found no evidence to support the notion that early thrombolysis might prevent recurrent PE or CTEPH, compared to anticoagulant therapy.

### Study limitation

The main limitation of this retrospective study was the small sample size. This limitation might have explained the lack of a significant difference in recurrent PE and CTEPH occurrences between the anticoagulant therapy group and the low-dose UK group. Future large-scale, multi-center, randomized controlled trials are needed to evaluate the efficacy and safety of low-dose UK treatment in intermediate-high-risk PE.

## Conclusion

The present study compared anticoagulation therapy to low-dose UK treatment for patients with intermediate-high-risk PE. More patients in the low-dose UK group showed rapid dissolution of intravascular thromboses and improved short-term and long-term dyspnea, compared to patients in the anticoagulation therapy group. However, we found no significant differences in mortality or in the incidences of recurrent PE and CTEPH between the two treatment groups. Additionally, low-dose UK treatment did not increase the risk of major or minor bleeding events, compared to anticoagulation therapy.

## Supporting information

S1 Data Data for all patients. (XLSX)

S1 File (DOCX)

S2 File (PDF)
