# Supplementary material for: Low-dose urokinase thrombolytic therapy for patients with acute intermediate-high-risk pulmonary embolism: A retrospective cohort study
Source: PLoS One. 2021 Mar 26;16(3):e0248603. doi: 10.1371/journal.pone.0248603 (PMC7997002; doi:10.1371/journal.pone.0248603)
Supplement: S1 File — (DOCX) [file pone.0248603.s002.docx]

**Fujian Provincial Hospital Ethics Committee**

**Clinical Research Trial Approval**

Ethics Committee Scientific Research No.(K2019-02-023)

| Approval project | Project Name | Low dose urokinase thrombolytic therapy for patients with acute intermediate-high-risk pulmonary embolism：A retrospective cohort study | | |
| --- | --- | --- | --- | --- |
|  | Project source | International cooperation projects * National scientific research projects* Provincial scientific research projects * Fuzhou scientific research projects * Research projects in hospital* Others✓ | | |
|  | Project number | / | Starting time and ending time | 2019.03-2020.08 |
|  | Department | Intensive care unit | Project Leader | Cuilian Weng |
|  | Title | associate chief physician | contact number | 13606031594 |
| Acceptance of review documents | Ethics Application Form  research plan | | | |
| Review methods | | Rapid review | | |
| Conclusion | According to the "Ethical review method of biomedical research involving human beings " (2016) issued by the Health and Family Planning Commission, the Food and Drug Administration's "Standards for quality management of drug clinical trials " (2003), the "Quality management standards for clinical trials of medical devices" (2016), "The declaration of Helsinki" (2013) of the World Medical Association, and the ethical principles of the International Committee of Medical Organizations "International Ethical Guidelines for Human Biomedical Research" (2002) , this study was reviewed by the ethics committee and agreed to carry out  the research according to the research plan. Yes No✓  Is the ethical review process ongoing 6 months 12 months | | | |
| Ethics committee stamp  2019-2-22 | | | | |

Address: Fuzhou Dongjie 134 Postcode:350001 Telephone :0591-88216023
