# Supplementary material for: Low-dose urokinase thrombolytic therapy for patients with acute intermediate-high-risk pulmonary embolism: A retrospective cohort study
Source: PLoS One. 2021 Mar 26;16(3):e0248603. doi: 10.1371/journal.pone.0248603 (PMC7997002; doi:10.1371/journal.pone.0248603)
Supplement: S2 File — (PDF) [file pone.0248603.s003.pdf]

福建省立医院伦理委员会

临床科研试验审批件

伦审科研第（K2019-02-023）**号**

<table>
<tr><td rowspan="5">审评项目</td><td>项目名称</td><td colspan="3">小剂量尿激酶溶栓治疗在中高危肺栓塞患者中有效性及安全性的回顾性队列研究</td></tr>
<tr><td>课题来源</td><td colspan="3">国际合作课题 ☐ 国家级科研课题 ☐ 省级科研课题 ☐
福州市科研课题 ☐ 医院科研课题 ☐ 其他 ☒</td></tr>
<tr><td>课题编号</td><td>/</td><td>起止时间</td><td>2019.03-2020.08</td></tr>
<tr><td>科室</td><td>重症医学科</td><td>课题负责人</td><td>翁翠莲</td></tr>
<tr><td>职称</td><td>副主任医师</td><td>联系电话</td><td>13606031594</td></tr>
<tr><td>受理审查文件</td><td colspan="4">伦理申请表
研究方案</td></tr>
<tr><td colspan="2">审查方式</td><td colspan="3">快速审查</td></tr>
<tr><td>结论</td><td colspan="4">根据卫计委《涉及人的生物医学研究伦理审查办法》(2016)、食品药品监督管理局《药物临床试验质量管理规范》(2003)、《医疗器械临床试验质量管理规范》(2016)、世界医学会《赫尔辛基宣言》(2013)、以及国际医学科学组织委员会《人体生物医学研究国际伦理指南》(2002)的伦理原则，经本伦理委员会审查，同意按照研究方案开展研究。
该研究进行过程中是否接受伦理委员会的持续审查 ☐是 ☒否
审查频度为研究批准之日起 ☐6个月 ☐ 12个月</td></tr>
<tr><td colspan="5">伦理委员会（盖章）：
2019年2月22日</td></tr>
</table>

地址：福州市东街134号 邮编：350001 电话：0591-88216023
